# Questions and answers on iron deficiency treatment selection and the use of intravenous iron in routine clinical practice

**DOI:** 10.1080/07853890.2020.1867323

**Published:** 2021-01-10

**Authors:** Toby Richards, Christian Breymann, Matthew J. Brookes, Stefan Lindgren, Iain C. Macdougall, Lawrence P. McMahon, Malcolm G. Munro, Elizabeta Nemeth, Giuseppe M. C. Rosano, Ingolf Schiefke, Günter Weiss

**Affiliations:** a Faculty of Health and Medical Sciences, University of Western Australia, Perth, Australia; b Obstetric Research-Feto Maternal Haematology Unit, University Hospital Zurich, Zurich, Switzerland; c Gastroenterology Unit, Royal Wolverhampton NHS Trust, Wolverhampton, UK; d Research Institute in Healthcare Science (RIHS), University of Wolverhampton, Wolverhampton, UK; e Department of Gastroenterology and Hepatology, Lund University, Skåne University Hospital, Malmö, Sweden; f Department of Renal Medicine, King’s College Hospital, London, UK; g Departments of Renal Medicine and Obstetric Medicine, Eastern Health Clinical School, Monash University, Melbourne, Australia; h Department of Obstetrics and Gynecology, David Geffen School of Medicine, University of California, Los Angeles, CA, USA; i Department of Obstetrics and Gynecology, Kaiser-Permanente, Los Angeles Medical Center, Los Angeles, CA, USA; j Center for Iron Disorders, David Geffen School of Medicine, University of California, Los Angeles, CA, USA; k Department of Medical Sciences, IRCCS San Raffaele, Roma, Italy; l Department of Gastroenterology, Hepatology, Diabetology and Endocrinology, Klinikum St. Georg, Leipzig, Germany; m Department of Internal Medicine II, Medical University Innsbruck, Innsbruck, Austria; n Christian Doppler Laboratory for Iron Metabolism and Anemia Research, University of Innsbruck, Innsbruck, Austria

**Keywords:** Anaemia, iron-deficiency, cardiovascular diseases, erythrocyte transfusion, inflammatory bowel diseases, infusions, intravenous, iron, menorrhagia, renal insufficiency, chronic, pregnancy complications

## Abstract

Iron deficiency is a common cause of morbidity and can arise as a consequence or complication from many diseases. The use of intravenous iron has increased significantly in the last decade, but concerns remain about indications and administration. Modern intravenous iron preparations can facilitate rapid iron repletion in one or two doses, both for absolute iron deficiency and, in the presence of inflammation, functional iron deficiency, where oral iron therapy is ineffective or has not worked. A multidisciplinary team of experts experienced in iron deficiency undertook a consensus review to support healthcare professionals with practical advice on managing iron deficiency in gastrointestinal, renal and cardiac disease, as well as; pregnancy, heavy menstrual bleeding, and surgery. We explain how intravenous iron may work where oral iron has not. We provide context on how and when intravenous iron should be administered, and informed opinion on potential benefits balanced with potential side-effects. We propose how intravenous iron side-effects can be anticipated in terms of what they may be and when they may occur. The aim of this consensus is to provide a practical basis for educating and preparing staff and patients on when and how iron infusions can be administered safely and efficiently.

**Key messages**
- Iron deficiency treatment selection is driven by several factors, including the presence of inflammation, the time available for iron replenishment, and the anticipated risk of side-effects or intolerance.
- Intravenous iron preparations are indicated for the treatment of iron deficiency when oral preparations are ineffective or cannot be used, and therefore have applicability in a wide range of clinical contexts, including chronic inflammatory conditions, perioperative settings, and disorders associated with chronic blood loss.
- Adverse events occurring with intravenous iron can be anticipated according to when they typically occur, which provides a basis for educating and preparing staff and patients on how iron infusions can be administered safely and efficiently.

## Introduction

The use of intravenous iron has grown substantially in the past decade due to heightened awareness of the impact of iron deficiency on clinical outcomes and quality of life. Current intravenous iron preparations are indicated for the treatment of iron deficiency when oral preparations are ineffective or cannot be used [1]. They have a wide range of applicability in clinical contexts including; nutritional deficiency, malabsorption, disorders associated with chronic blood loss, and chronic inflammatory conditions [2–5]. The availability of modern preparations allows rapid iron repletion in just one or two doses facilitating ease of treatment [6,7].

Intravenous iron preparations are approved with broad labels and the clinical trials testing efficacy span many indications and disease groups, this has led to a proliferation of guidelines and consensus statements. However, there remains a need for more practical “how to” guidance on intravenous iron administration as patients and staff may express concerns over potential side-effects, adverse events, reactions and complications, or contra-indications, which may be confused or misperceived.

Here, we aim to support healthcare professionals by summarizing the current recommendations for intravenous iron, alongside practical advice on their administration and informed opinion on the anticipated risks versus expected benefits of treatment.

## What is iron deficiency?

Iron is an essential trace mineral that plays a role in vital organismal and cellular processes including (but not limited to) the transport of oxygen by haemoglobin (Hb), mitochondrial energy metabolism, and a large number of enzymatic processes across multiple tissue sites [8,9]. Prolonged iron deficiency restricts Hb synthesis in the marrow and results in iron deficiency anaemia. In addition to affecting erythropoiesis, iron deficiency also influences the function of other cells and tissues, impairing myocardium and skeletal muscle, the immune system (and T-cell activity in particular), as well as cognitive and neuronal development [10].

“Absolute” or “true” iron deficiency develops as a result of an imbalance between iron uptake, iron utilization and iron loss [11]. This may occur due to acute or chronic blood loss, sustained limitation in dietary iron absorption, or because of inadequate iron supply during periods with increased iron requirements (e.g. rapid growth, pregnancy, and erythropoiesis-stimulating therapy). Absolute iron deficiency is manifest as a decrease in total body iron, primarily evident in decreased iron stores in the liver and spleen, as reflected in low serum levels of the iron storage protein, ferritin. The changes in serum ferritin levels, however, occur only when iron storage has been largely depleted.

Functional iron deficiency describes a state of impaired iron mobilization that arises in conditions associated with inflammation, infection or chronic disease, where the normal pathways of iron transport and iron metabolism are disrupted [11,12]. In these settings, cytokines, particularly IL6, upregulate the master regulator of iron homeostasis, hepcidin. Increased hepcidin (normally seen in response to iron loading) leads to reduced dietary iron absorption and failure of cellular iron export into plasma, with retention of iron within macrophages. Consequently, this reduces circulating levels of iron and its availability for erythropoiesis despite the presence of adequate iron stores.

## What is the difference between iron deficiency anaemia and anaemia of chronic disease?

Definitions of iron deficiency are frequently inconsistent because absolute and functional iron deficiency are often used interchangeably. Absolute iron deficiency is commonly defined by reduced ferritin levels (<30 µg/L), with low circulating iron and reduced binding of iron to the transport protein transferrin, as measured by low transferrin saturation (TSAT; <20%)[12]. Prolonged absolute iron deficiency leads to the emergence of iron deficiency anaemia (IDA), defined according to World Health Organization (WHO) criteria as Hb <120 g/L in women (<110 g/L in pregnant women) and <130 g/L in men (although these levels represent lower limits of normal Hb – levels are typically much higher in healthy individuals). IDA is typically characterized by microcytic and hypochromic erythrocytes [11,13].

Functional iron deficiency is defined by low circulating iron levels and TSAT <20% but with normal or increased serum ferritin levels (>100 µg/L) along with systemic markers of inflammation (C-reactive protein [CRP] and interleukin 6 [IL-6]) [12]. Over time, the presence of functional iron deficiency and the additional effects of immune mediators on erythropoiesis lead to the development of anaemia of chronic disease (sometimes also referred to as anaemia of inflammation), which is mostly normochromic and normocytic [12].

**Figure 1.**
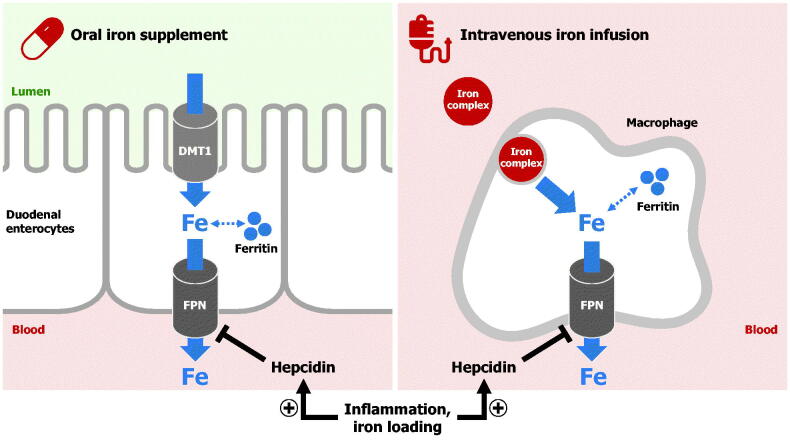
The mechanism of oral and intravenous iron treatments. Iron administered via an oral iron supplement is absorbed by duodenal enterocytes. Divalent metal transporter 1 (DMT1) imports iron across the apical surface of enterocytes, whereas ferroportin exports iron across the basolateral surface. The hormone hepcidin, which is increased by iron loading or inflammation, impairs cellular iron export into blood by causing ferroportin degradation. Intravenous iron preparations are administered by infusion, and the iron‒carbohydrate complex is taken up and processed by macrophages in the liver, spleen and marrow. Once iron is released into the cytoplasm, it is either stored in ferritin or exported from macrophages through ferroportin (FPN). Intravenous iron can overcome the hepcidin-mediated block of iron absorption from the gut.

Absolute iron deficiency is the most common cause for anaemia worldwide due to low nutritional iron intake or excess blood loss. Functional iron deficiency is the second most common cause for anaemia worldwide and is often found in patients suffering from infections, autoimmune diseases, cancer, congestive heart failure, obesity or chronic obstructive pulmonary disease [12]. In addition, patients with chronic kidney disease (CKD) also have features of functional iron deficiency with increased circulating hepcidin levels due to subtle inflammation and often impaired urinary hepcidin excretion [14] (Figure 1).

Current guidelines typically suggest that a serum ferritin level below 100 µg/L is considered indicative of absolute iron deficiency in the setting of an inflammatory disease. Alternative laboratory determinates include; soluble transferrin receptor (sTfR) levels, sTfR/log ferritin ratio, percentage of hypochromic red blood cells, reticulocyte Hb content, hepcidin, and red cell indices may be helpful to identify absolute iron deficiency in patients with ferritin levels >100 µg/L, but these are mostly used in academic settings [15,16]. The use of hepcidin levels for diagnostic purposes or to guide efficacy of therapeutic iron supplementation is not currently validated in clinical practice [17].

## How does iron repletion differ with oral versus intravenous iron?

The treatment goal is to refill iron stores and in cases of anaemia, normalize Hb concentration. Oral iron supplements are absorbed across the small intestinal epithelium, mainly in the duodenum, *via* iron transporters (DMT1 and ferroportin) on the apical and basolateral surface of enterocytes (Figure 1). Only ∼10% of intestinal iron is absorbed on average [18]. Thus, of the common therapeutic oral dose of 60–180 mg elemental iron, less than 20 mg is absorbed per day, meaning that theoretically 10–30 days of continuous iron supplementation may be required to achieve a 10 g/L increase in Hb, and as long as 6 months to fully normalize Hb levels and replenish iron stores in anaemic patients. When given orally, residual iron supplement remains largely unabsorbed in the digestive tract, which can injure intestinal surfaces and alter the composition of the gut microbiome, leading to gastrointestinal side-effects.

Intravenous iron preparations bypass gastrointestinal absorption and their iron–carbohydrate complexes are processed by macrophages in order to release iron [19]. This is primarily performed by macrophages in the liver, spleen and bone marrow, but the mechanism of the uptake and subsequent degradation of the complex is incompletely understood. Once iron is released into the cytoplasm, it can be stored within ferritin or exported by ferroportin into plasma (Figure 1). Depending on the specific intravenous iron preparation, the total amount of iron that can be administered through a single infusion may range from 62.5 mg to >1500 mg. Therefore, for those preparations delivering a large amount of iron, even a single dose can be sufficient for the complete replacement of iron deficit in patients.

Ferroportin, the transporter required for iron export into plasma from both duodenal enterocytes and macrophages, is regulated by the hormone hepcidin (Figure 1). Hepcidin occludes and degrades ferroportin; hence, elevated hepcidin impairs oral iron absorption and decreases the release of iron from macrophages [20]. Hepcidin is homeostatically regulated by multiple signals, including circulating iron and erythropoietic factors. In iron deficiency, hepcidin levels are low, allowing efficient export of iron from enterocytes and macrophages. During iron loading or in inflammatory conditions, hepcidin is elevated and prevents iron flow into plasma. Theoretically, intravenous iron may partially overcome the hepcidin-mediated block of macrophage iron release by increasing intracellular iron levels, which stimulates macrophage ferroportin translation *via* the iron responsive element–iron regulatory protein (IRE–IRP) system [21].

## When should iron therapy be used in inflammatory bowel disease?

Around two in three inpatients and one in six outpatients with inflammatory bowel disease (IBD) have IDA [22], and an even higher proportion have iron deficiency in the absence of overt anaemia. This causes fatigue and impacts cognitive function and patientquality of life. Oral iron should not be used in patients with active IBD because systemic inflammation (and thus elevated hepcidin) negates its effectiveness, and, additionally, these preparations can exacerbate IBD activity in the bowel. In patients with inactive IBD and iron deficiency, we recommend that no more than 100 mg elemental iron should be taken daily and absorption may be improved by once daily or alternate daily dosing regimens [23,24].

It is important to consider that in the assessment of iron deficiency in IBD, ferritin levels up to 100 μg/L in the presence of inflammation may still reflect absolute iron deficiency [23]. Ascertainment of iron status in such cases is problematic, although other markers of iron metabolism can be used as outlined above. It is also important to consider other rare causes of anaemia in patients with IBD (e.g. folate deficiency and vitamin B12 deficiency, or drug-induced bone marrow depression). After treatment, follow-up is mandatory to avoid recurrence of iron deficiency, particularly in active IBD and patients may require in excess of 3000 mg annually.

In summary, we recommend that intravenous iron should be used first line in the following IBD patient groups: (1) those with active inflammation; (2) those with moderate to severe anaemia (Hb <100 g/L); (3) those with intolerance to oral iron, including a previous history of intolerance; and (4) those who need erythropoiesis-stimulating agents (ESAs) [23].

## When should iron therapy be used in CKD?

Intravenous iron is used routinely in nearly all patients requiring haemodialysis (HD), and is variably required in patients with non-dialysis-dependent CKD, those on peritoneal dialysis (PD), and renal transplant recipients [4].

In patients with non-dialysis-dependent CKD (estimated glomerular filtration rate [eGFR] ≤ 60 mL/min/1.73 m^2^) the FIND-CKD study showed that targeting a high ferritin level (400–600 μg/L) with intravenous iron was more successful in increasing Hb levels than oral iron and may contribute to improved overall anaemia management [25].

Among those on dialysis, PD patients do appear to benefit from supranormal iron indices, with a significant rise in Hb following intravenous iron [26]. Many patients with minimal ongoing iron losses will require bolus infusions of up to 500 mg of intravenous iron, but some patients who may also have heavy menstrual bleeding and/or higher gastrointestinal blood loss may require periodic infusions of 1000 mg or more.

In HD patients, higher iron indices reduce the required dose of ESAs needed to achieve an adequate Hb response [27]. For patients on HD, either bolus infusions (up to 1500 mg) or incremental dosing (usually 100‒200 mg) can be given during dialysis. Recently, a large randomized trial by Macdougall IC et al. [28] suggested some haematopoietic and cardiovascular benefits by administering 400 mg of intravenous iron sucrose monthly, stopping temporarily if the serum ferritin exceeded 700 µg/L or the transferrin saturation was ≥40%. Concerns regarding increased mortality from infection and organ toxicity were not realized in this event-driven study [28–30].

Patients with a stable kidney transplant exhibit a more marked fall in Hb as their renal function decreases compared with the (non-immunosuppressed) general population; however, the need for intravenous iron and its relationship to the dose of ESA administered in this group is uncertain. Many physicians will manage anaemia in this population in a similar manner to other non-dialysis-dependent CKD patients.

In summary, we recommend that intravenous iron should be used as follows:

- First-line in haemodialysis patients in order to optimize iron stores, especially in the context of concurrent ESA use.
- For end-stage kidney disease patients receiving peritoneal dialysis and for non-dialysis patients with advanced (stage 4–5) CKD, intravenous iron should be a ready therapeutic option.
- For less-advanced (stage 1–3) CKD, oral iron supplementation is acceptable as a first-line option. However, intravenous iron may become necessary for patients who are poorly responsive or who develop troublesome gastrointestinal side-effects.

## When should iron therapy be used in heart failure?

Iron deficiency reduces skeletal and cardiac muscle function and therefore significantly affects functional capacity and prognosis of patients with heart failure. Up to 83% of acute heart failure (HF) patients and 35–55% of chronic HF patients have iron deficiency, which confers an increased risk of adverse outcomes, independent of concurrent anaemia [31,32]. All patients with HF should be periodically screened for iron deficiency, and the current European Society of Cardiology (ESC)/Heart Failure Association (HFA) guidelines on HF recommend inclusion of serum ferritin and TSAT tests in the initial assessment of newly diagnosed patients [5]. The presence of iron deficiency should prompt further investigation to identify its cause and treatment.

Randomized studies and meta-analyses have shown that intravenous iron has a good safety profile and improves outcomes, including symptoms, exercise capacity and quality of life [33–37]; these benefits are independent of anaemia coexistence [38]. One randomized study and one individual-patient meta-analysis showed a reduction in HF hospitalizations and a reduced composite outcome of recurrent cardiovascular hospitalizations and cardiovascular mortality, respectively [34,38]. A recent outcomes study has generated further evidence on the benefits of intravenous iron supplementation in patients with iron deficiency and LVEF <50% who are stabilized after an episode of acute HF; reporting a reduction in the risk of subsequent HF hospitalizations but with no apparent effect on the risk of cardiovascular death [39].

Oral iron supplementation failed to improve exercise capacity in HF patients with iron deficiency [40]. Ongoing randomized controlled clinical trials are expected to provide further evidence regarding the effects of intravenous iron supplementation on survival and long-term safety in patients. Based on current data, intravenous iron should be considered in iron deficiency defined as serum ferritin <100 µg/L or serum ferritin 100‒299 µg/L with TSAT <20%.

For patients with anaemia in HF, early trials with relatively small sample sizes initially suggested that erythropoietin may have beneficial effect [41]. However, in the only large-scale randomized trial, darbepoetin alfa did not improve prognosis in HF patients with reduced ejection fraction and mild to moderate anaemia, but did increase the risk of thromboembolic events [42]. Treatment of anaemia in HF with ESAs is contraindicated in the absence of other indications for this therapy.

In summary, we recommend that intravenous iron should be considered in iron-deficient HF patients.

## When should iron therapy be used during and after pregnancy?

The WHO estimates that the worldwide prevalence of anaemia in pregnancy ranges from 23% in industrialized countries to 52% in non-industrialized countries [43]. The incidence of iron deficiency without anaemia is not known, but low iron levels in the first trimester predict onset of anaemia at term [44]. Postpartum anaemia continues to be among the commonest causes of death in women who have just given birth in developing countries, [45] and a common cause for transfusion in high income countries [46].

Oral iron can be used for the prevention of iron deficiency during pregnancy for women with reduced iron reserves (i.e. ferritin <30 µg/L), mild anaemia (i.e. Hb >100 g/L), and during the first trimester when intravenous iron is contraindicated [47]

Intravenous iron should be used from the second trimester onwards in cases of severe iron deficiency anaemia (i.e. Hb <90 g/L), when patients are intolerant or non-responsive to oral iron, or when there is a clinical need for rapid and efficient anaemia treatment [47]. Of note, intravenous iron administration leads to greater increases in maternal Hb levels and iron stores compared with oral iron [48].

In postpartum anaemia (i.e. Hb <100 g/L), intravenous iron is superior to oral iron in treating both anaemia and iron deficiency [49] with positive effects on quality of life parameters, fatigue and social functioning [50].

In summary, we recommend that intravenous iron should be used in the following pregnant patient groups:

- Pregnant women with moderate anaemia (Hb 95–105 g/L) who do not respond to oral iron after 4 weeks of intake.
- Pregnant women with severe anaemia (Hb <95 g/L) as a first line option.
- Pregnant women with moderate to severe anaemia close to birth, i.e. 4 weeks
- Pregnant anaemic women (Hb <105 g/L) with high risk for high blood losses and blood transfusion, i.e. placenta previa, clotting abnormalities, uterine fibroids and others.
- Pregnant women with intolerance to oral iron in case of anaemia.
- Women with postpartum anaemia (Hb <100 g/L).

## When should iron therapy be used in women with heavy menstrual bleeding?

Heavy menstrual bleeding (HMB) is a symptom that should be defined by patients themselves as “…excessive menstrual blood loss which interferes with physical, social, emotional and/or material quality of life” [51,52]. Women can be unaware that their menstrual bleeding is abnormal [53–55], while others suppress symptoms [56] or present to providers who seem to normalize the symptoms, which remain untreated [57,58]. Iron deficiency and anaemia is common in this population [1]

Management of women with the symptom of HMB and associated iron deficiency, with or without anaemia, requires dual therapy – interventions directed at the cause of the HMB and treatment designed to correct the iron deficiency and anaemia.

Causes of abnormal uterine bleeding in the reproductive years are classified by PALM-COEIN: Polyps, Adenomyosis, Leiomyoma, Malignancy and hyperplasia, Coagulopathy, Ovulatory disorders, Endometrial causes, Iatrogenic, and Not otherwise classified) [59,60]. Interventions require a structured approach to diagnosis and determination of appropriate options with consideration of; costs, anticipated effectiveness, associated morbidity, desire for future fertility, cultural norms, and personal preferences.

Treatment of iron deficiency with oral iron therapy should be the initial approach. Unfortunately, 20–40% of non-pregnant women ingesting oral iron preparations experience nausea, vomiting, constipation and/or an undesirable metallic taste, and often stop oral iron therapy [1,61]. These side-effects may be reduced with changes in formulation, the addition of stool softeners for constipation, or by alternate-day administration.

With this in mind, women should be reassessed and failure of treatment (defined by Hb rise <10 g/L in one month or ferritin <30 µg/L at 3 months) should trigger the use of intravenous iron, which has seen a considerable increase in use in this setting over the last decade [62].

In summary, we recommend that intravenous iron should be used in girls and women with iron deficiency anaemia:

1. Who do not tolerate or are inappropriate for the use of oral iron.
2. Who have failed to respond to an appropriate dose, formulation and schedule of oral iron administration within 30 days as defined by an increase in Hb of at least 10 g/L (or 1 g/L).
3. Who are scheduled for elective gynaecologic surgery and who, in the opinion of the surgeon, are unlikely to achieve a Hb of at least 110 g/L (11 g/dL) by the day of surgery using oral formulations.

## What are the risks of intravenous iron and how should they be managed?

Overall the rates of drug-related adverse events with intravenous iron are extremely low, occurring at a rate of approximately 38 events per million doses, as reported in a historical analysis of United States Food and Drug Administration(FDA) data [63]. Serious hypersensitivity reactions (anaphylaxis) with modern intravenous iron are now rare, occurring at a rate of 24 per 100,000 persons (95% CI, 20.0–29.5) for non-dextran intravenous iron products at first exposure in a large observational studyof non-dialysis USMedicare patients (*n* = 688,183) [64]. Overall, results comparable to most other intravenous drugs and therapies [65].

A meta-analysis of 103 trials involving intravenous iron therapy conducted by the Mayo Clinic found no increased risk of serious adverse events compared with controls (relative risk [RR], 1.04; 95% confidence interval [CI], 0.93‒1.17) [66].

### Recommendations on the administration of intravenous iron

The indications and reasons for an iron infusion should be explained clearly to patients following a full consultation and examination. The indication for intravenous iron should be confirmed and a plan made to address the underlying cause including investigation and management for any cause for bleeding where appropriate.

We would also recommend that patients have time to read an information sheet or online resource independently, prior to their treatment visit, which should include the potential risks of side-effects or complications that patients may experience. Consent for intravenous iron should be documented.

**Figure 2.**
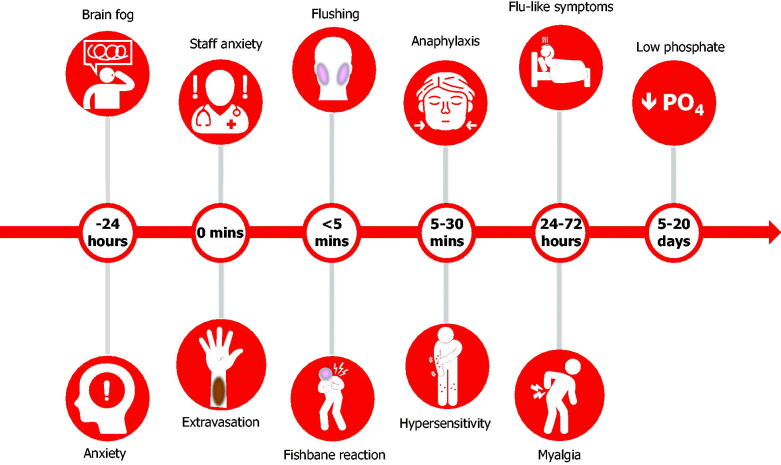
Adverse events associated with the administration of intravenous iron. Adverse events occurring with intravenous iron can be anticipated according to when they typically occur. Educational material and institutional training should prepare patients and staff for their occurrence, minimizing the need to unnecessarily withhold or abandon administration and reducing the need for subsequent patient visits.

The preparation, dosing and administration of intravenous iron varies between products and should be checked against corresponding prescribing information and country-specific guidance. Administration should always take place in appropriately equipped practices, clinics or infusion centres. Most adverse events occurring with intravenous iron can be anticipated by their nature and according to when they typically occur (Figure 2). Recommended strategies to manage common events are described below.

1. **Anxiety:** Any intervention is a concern for patients. This is normal in most scenarios but may be exacerbated since iron deficiency itself can affect cognitive function and cause physical symptoms such as anxiety, palpitations, chest pain and shortness of breath.
  - Careful explanation combined with empathetic well-trained staff in a well-organized and relaxed environment can help ensure patients are reassured and able to undergo treatment [67].
  - Appropriate training of supporting staff can also reduce the chances of their own anxiety being transferred to patients.
2. **Tattooing:** Staining can occur from iron either within the vein itself or more significantly from extravasation of iron, leading to tattooing [68].
  - In preparation, ensure patients are well hydrated and warm before attempting the intravenous cannula (a large hot drink is an easy solution).
  - In difficult cases, consider using ultrasound-guided cannula placement.
  - The intravenous cannula should be flushed with a minimum of 10 mL N/saline (or even a separate 100 mL bag of saline)to confirm placement before the infusion starts. The cannula should be well secured and checked during the infusion. At completion, disconnect the iron infusion and undertake a further10 mL N/saline flush to ensure there is no residual iron in the cannula that could leak out and stain the vein or skin on removal.
  - Stains may resolve over time, but this can take up to 2 years and is not guaranteed, therefore referral to dermatology for laser therapy may be considered [69].
3. **Flushing and Fishbane reaction:** An initial slower rate of infusion is recommended over the first couple of minutes of an intravenous iron administration as some patients may experience flushing with associated light-headedness, dizziness or nausea. This is believed to be caused by unbound labile iron interacting with the endothelium leading to the release of nitric oxide, referred to as a “Fishbane reaction” [70]. These acute effects are self-limiting, typically lasting a couple of minutes.
  - Management is to stop the infusion for several minutes, reassure the patient, and provide a glass of water. The iron infusion can then be restarted slowly and usually completed.
  - Patients should made aware of the possibility of flushing reactions prior to administration and advised to arrive well hydrated.
  - Intravenous irons should also be prepared in the prescribed volume of saline (100–250 mL) and not over diluted as this may increase the free or labile iron during infusion.
4. **Hypersensitivity reactions:** The rate of hypersensitivity reactions with intravenous iron is less than 0.1% [71]; the risk is enhanced for patients with known allergies, a history of severe asthma, eczema or other atopic allergy, and patients with immune or inflammatory conditions. In this latter high-risk group, premedication with a steroid injection can be considered (e.g. hydrocortisone 200 mg), we would not suggest intravenous Piriton (chlorphenamine maleate) as the side-effects of the medication are often mistaken or worse than any effects of the iron infusion [72,73]. From a clinical perspective, reactions may start within seconds of commencing the infusion and often be confused with a flushing reaction. Some hypersensitivity reactions can occur in the30-minute period of observation after completion of the infusion (so it is advisable to leave the cannula in situ). Diagnosis and treatment depends on the relevant clinical picture and the severity of the response. In most cases, hypersensitivity reactions are limited with flushing, itching and urticarial rash.
  - Management in mild cases is to stop the infusion and monitor the patient as symptoms pass and settle in about 10 minutes, after which time the infusion can be recommenced at a reduced rate and finished (providing no further symptoms occur) [74,75].
  - In moderate cases, a steroid injection can be considered (e.g. hydrocortisone 200 mg), with or without a 500 mL fluid bolus according to the patient’s observations. Patients who experience symptoms should be monitored for further progression.
  - Serious hypersensitivity reactions (anaphylaxis) are rare and typically characterized by a sudden onset of symptoms or progressive worsening in some cases. Extensive guidance on risk minimization and protocols for management of hypersensitivity reactions were published by other consensus groups [76,77]. Staff should be familiar with these protocols and trained in the management of severe hypersensitivity reactions including access to resuscitation facilities.
5. **Post-infusion flu:** Patients often report flu-like symptoms 2–5 days after receiving an iron infusion. These include; myalgia, aching, bone pain and, in some cases, increased temperature [78,79]. These types of symptoms may be more common than most institutions document, affecting up to one-third of all patients [80]. Symptoms are self-limiting and typically last 24–48 hours; however, this can be alarming to affected patients. The condition should not be confused with an “allergic reaction” or hypersensitivity, which is rare once an infusion is completed.
  - Patients should be reminded of the possibility of symptoms before leaving the institution and advised to stay well hydrated and take ibuprofen if needed.
6. **Hypophosphataemia:** One potential consequence of intravenous iron recognized recently is a fall in serum phosphate with several formulations, particularly ferric carboxymaltose [81]. Concentrations fall below the normal range in up to 40% of cases and relate to release of a hormone, fibroblast growth factor-23 (FGF-23), from osteocytes [81–83]. This prompts receptor-mediated renal phosphate excretion, a reduction in circulating parathyroid hormone levels, and indirectly reduces gut phosphate absorption through an FGF-23-mediated reduction in 1,25-di-OH vitamin D [83]. The effect is usually asymptomatic and reaches a nadir between 1 and 2 weeks [81]. In most cases, phosphate concentrations have returned to baseline by 12 weeks [82,84]. Isolated case reports, often in the setting of other metabolic disorders and mostly in patients receiving multiple infusions, have shown a potential association between longer periods of hypophosphataemia and osteomalacia and fractures [83,85]. These events are extremely rare and there are currently no intravenous iron-related clinical trial data that report an association between clinical adverse events and low phosphate levels [82,86].
  - Patient management should be based on an assessment of risk.
  - There is a strong consensus that patients with IBD should be checked for micronutrient deficiencies on a regular basis (regardless of iron administration), with specific deficits appropriately corrected [3,87].
  - Monitoring of serum phosphate levels may be indicated in patients at risk for low serum phosphate who require a repeat course of treatment [88,89].

In summary, clarification over the sequence of events and likely risks following an iron infusion is important for both staff and patients to ensure that intravenous iron is administered in the safest manner for patient benefit. Given that iron deficiency and the acute but non-life-threatening side-effects of intravenous iron are both associated with an array of generalized symptoms, it is possible that concerns over hypersensitivity and hypophosphataemia may be misconstrued with more common symptomatic events, such as anxiety, Fishbane reactions, and post-infusion flu-like symptoms. Reassurance and knowledge of the clinical trial data are important, where adverse events are well-documented and independently adjudicated, to inform staff and patients of the benefits and potential risk of intravenous iron.

## Conclusion

Intravenous iron repletion is a transformative therapy that can have a major bearing on clinically relevant outcomes and overall well-being. Treating physicians should always aim to establish the underlying cause of bleeding, address it with appropriate therapy when possible, and understand the impact of inflammation and chronic disease on iron deficiency. Intravenous iron has a significant role to play in patients’ health but it is important to be well informed of potential adverse events and timing thereof to help staff and patients ensure treatment is administered safely and efficiently.
